# Prognostic Significance of Lymph Node Examination by the OSNA Method in Lung Cancer Patients—Comparison with the Standard Histopathological Procedure

**DOI:** 10.3390/cells9122611

**Published:** 2020-12-04

**Authors:** Josef Vodicka, Martin Pesta, Vlastimil Kulda, Katerina Houfkova, Bohuslava Vankova, Jakub Sebek, Martin Skala, Jakub Fichtl, Kristyna Prochazkova, Ondrej Topolcan

**Affiliations:** 1 Department of Surgery, Faculty Hospital Plzen, Faculty of Medicine in Pilsen, Charles University, alej Svobody 80, 304 60 Plzen, Czech Republic; vodicka@fnplzen.cz (J.V.); sebekj@fnplzen.cz (J.S.); skalama@fnplzen.cz (M.S.); fichtlj@fnplzen.cz (J.F.); prochazkovak@fnplzen.cz (K.P.); 2 Department of Biology, Faculty of Medicine in Pilsen, Charles University, alej Svobody 76, 323 00 Plzen, Czech Republic; katerina.houfkova@lfp.cuni.cz; 3 Biomedical Center, Faculty of Medicine in Pilsen, Charles University, alej Svobody 76, 323 00 Plzen, Czech Republic; 4 Department of Medical Chemistry and Biochemistry, Faculty of Medicine in Pilsen, Charles University, Karlovarska 48, 301 66 Plzen, Czech Republic; vlastimil.kulda@lfp.cuni.cz; 5 Department of Pathology, Faculty Hospital Plzen, Faculty of Medicine in Pilsen, Charles University, Edvarda Benese 13, 305 99 Plzen, Czech Republic; kokoskovab@fnplzen.cz; 6 Department of Immunochemistry Diagnostics, Faculty Hospital Plzen, Faculty of Medicine in Pilsen, Charles University, Edvarda Benese 13, 305 99 Plzen, Czech Republic; topolcan@fnplzen.cz

**Keywords:** lung cancer, lymph nodes, H&E, IHC CK19, OSNA

## Abstract

The aim of the study was to compare the prognostic significance of lymph node status of patients with lung cancer analyzed by three different methods: hematoxylin and eosin (H&E), immunohistochemistry of cytokeratin 19 (IHC CK19), and One-Step Nucleic Acid Amplification (OSNA). The clinical relevance of the results was evaluated based on relation to prognosis; the disease-free interval (DFI) and overall survival (OS) were analyzed. During radical surgical treatment, a total of 1426 lymph nodes were obtained from 100 patients, creating 472 groups of nodes (4–5 groups per patient) and examined by H&E, IHC CK19 and OSNA. The median follow-up was 44 months. Concordant results on the lymph node status of the H&E, IHC CK19 and OSNA examinations were reported in 78% of patients. We recorded shorter OS in patients with positive results provided by both OSNA and H&E. The study demonstrated a higher percentage of detected micrometastases in lymph nodes by the OSNA method. However, the higher sensitivity of the OSNA, with the cut-off value 250 copies of mRNA of CK19/µL, resulted in a lower association of OSNA positivity with progress of the disease compared to H&E. Increasing the cut-off to 615 copies resulted in an increase in concordance between the OSNA and H&E, which means that the higher cut-off is more relevant in the case of lung tumors.

## 1. Introduction

Today, the standard detection of tumor cells in hilar and mediastinal lymph nodes (LNs) removed during radical surgical treatment of primary and secondary lung tumors is performed using histopathological methods, i.e., by the microscopic examination of specimens stained using hematoxylin–eosin (H&E) by pathologists. However, this approach is limited, in particular in the case of micrometastases, clusters (clusters of tumor cells), or isolated tumor cells, where, according to certain studies, false negative results of the examination are produced in up to 20% of cases [1]. One of the available methods for the improved detection of micrometastases in regional LNs can be, based on data provided by recent studies, both the proof of tumor cells by immunohistochemical examination with an antibody against cytokeratin 19 (IHC CK19), and the molecular genetic method OSNA (One-Step Nucleic Acid Amplification), which detects CK19 mRNA [2,3,4]. Although the IHC CK19 method has been available for a number of years, it has not yet been routinely used for the histopathological examination of LNs. The molecular genetic method OSNA represents a more recent approach to the proof of CK19 in LNs, based on detecting CK19 mRNA copies in a given sample by isothermal amplification (LAMP).

A number of studies comparing the OSNA method with the standard histopathological examination in patients with breast cancer and/or colorectal carcinoma (CRC) have been published. The conclusions of these studies presented comparable results (concordance level of 95–97%, OSNA to H&E sensitivity 86–96%, OSNA to H&E specificity 92–100%) [1,2,4,5,6,7,8,9,10]. Recently, there are efforts to implement LN examination by OSNA method in management of patients with other tumors, e.g., endometrial cancer [11], gastric cancer [12] or thyroid carcinoma [13,14].

In the case of lung tumors, the OSNA method has been the subject of several studies, the purpose of which was both to ascertain the presence of CK19 in these tumors and offer a comparison of OSNA to H&E [3,15,16,17]. Among them, was also our prospective study commenced in 2015, the purpose of which was to refine the pathological TNM (pTNM) lymph node staging (N0, N1, N2) of primary and secondary lung tumors by the more sensitive detection of micrometastases in LNs using the IHC CK19 and the OSNA method compared to the H&E examination, specifically by a thorough analysis of all intra-operatively removed LNs. In 2018, we published the results of the comparison of the OSNA, H&E, and IHC CK19 methods, which were based on a set of 64 patients (885 examined nodes) [18]. The results showed a higher percentage of detected micrometastases in hilar and mediastinal lymph nodes when examined by the OSNA method compared to H&E and IHC CK19 (upstaging by 16%). In addition, we proposed a method for the clinical application of the OSNA method to lung tumors based on the pooling of LNs [18].

The aim of the present study was to analyze, by carefully monitoring patients, the relationship between micrometastases detected in LNs using the OSNA, H&E, and IHC CK19 methods and the progress of the disease (the median follow-up was 44 months) in a group of 100 patients.

## 2. Materials and Methods

### 2.1. Patients

The inclusion of patients with operable non-small cell lung carcinoma (NSCLC) and pulmonary metastases of colorectal carcinoma in the prospective study was performed in the period of 2015–2017. The study was approved by the Ethics Committee of the Faculty Hospital in Pilsen (No. 20150604). All patients provided their informed consent for inclusion in the study. The detailed characteristics of the group of 100 patients are provided in Table 1 (80 patients with NSCLC) and Table 2 (20 patients with pulmonary metastases of colorectal carcinoma). In all patients, the primary pulmonary tumor, or pulmonary metastasis, was radically removed and in all cases a systematic nodal dissection was executed according to the well-proven scheme of the International Association for the Study of Lung Cancer (IASLC) of 2009 [19]. The patients’ median follow-up was 44 months.

### 2.2. Examination of Lymph Nodes

During the surgery of 100 patients, a total of 1426 lymph nodes were removed, with the average value being 14.3 LNs/person (5–32 LNs). Each LN was dissected into four parts, whereby parts one and three were examined using the H&E and IHC CK19 method, and parts two and four were examined using the OSNA assay. For methodological reasons, parts of LNs for OSNA method (Sysmex, Kobe, Japan) were pooled into 3–5 groups of LNs within the framework of one nodal zone based on the IASLC LNs classification developed from the Mountain–Dresler classification [19] creating 472 groups in total. On the basis of the results on groups of nodes, we obtained lymph node staging for a patient (N0, N1, N2).

The removal of lymph nodes, the scheme for dissecting and pooling, as well as the histopathological and immunohistochemical examinations, CK19 detection and the OSNA method, are described in detail in our previous article [18].

Briefly, LNs of one group were homogenized and lysed according to the manufacturer’s instructions. Determination was performed using the diagnostic kit LYNOAMP BC OSNA (Sysmex, Kobe, Japan). Determination of the presence of mRNA CK19, as the marker of epithelial cells, was executed using the RD100i instrument (Sysmex, Kobe, Japan). The net time of amplification reaction is 16 minutes, up to four groups of pooled lymph nodes can be analyzed in parallel. The cut-off values for the OSNA method were determined as specified in the LYNOAMP BC OSNA manufacturer’s manual and were used in the previous studies of tumor cells in LNs in patients with lung cancer (absence of tumor cells—cut-off value < 250 copies of mRNA of CK19/µL, presence of a micrometastasis—cut-off value ranging from 250 to 5000 copies of mRNA of CK19/µL, presence of a macrometastasis—cut-off value > 5000 copies of mRNA of CK19/µL) [2,3,15,20,21].

### 2.3. Statistical Analysis

The statistical analysis was calculated using the SAS software (SAS Institute Inc., Cary, NC, USA). Essential descriptive statistics for all variables of interest were prepared based on the clinical and pathological data of the patients. Categorical variables were reported as absolute and percentage values. Venn diagrams were used for comparing the results of H&E, IHC CK19 and OSNA method. Kaplan–Meier survival curves for overall survival (OS) and disease-free interval (DFI) were generated to compare prognostic significance of results obtained by H&E and OSNA method. The statistical significance of differences in CK19 mRNA copies assessed by OSNA between H&E-positive and H&E-negative samples was calculated using t test for independent samples. The statistical significance level was determined at the alpha limit = 5%.

## 3. Results

### 3.1. Concordance among H&E, IHC CK19 and OSNA for Groups of LNs

**Figure 1:**
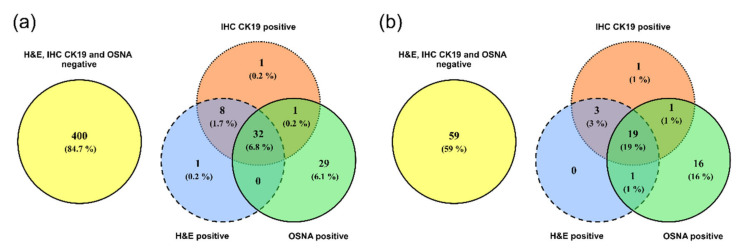
Comparison of examination results for the presence of tumor cells using the H&E, IHC CK19 and OSNA methods by Venn diagrams. (**a**) Groups of lymph nodes; (**b**) patients.

The concordance of the H&E, IHC CK19 and OSNA examinations was evaluated in 472 groups of lymph nodes. Full concordance of the three methods was recorded in 432 groups of lymph nodes (91.5%), 400 groups of lymph nodes were concordantly negative, and 32 groups of lymph nodes were concordantly positive, see Figure 1a.

### 3.2. Concordance among H&E, IHC CK19 and OSNA for Individual Patients

After evaluating the groups of lymph nodes for a given patient (in each patient, 3–5 groups of lymph nodes were evaluated), concordance was recorded in 78 patients (78%), of whom 59 patients had all examined LNs concordantly negative and 19 patients concordantly positive. Micrometastases in the lymph nodes were detected in 16 patients (16%) by the OSNA method, with negative results for the other methods. In three cases (3%), the H&E and IHC CK19 examinations were positive while the OSNA method produced negative results, see Figure 1b.

### 3.3. Comparison of Prognostic Significance of H&E and OSNA

**Figure 2:**
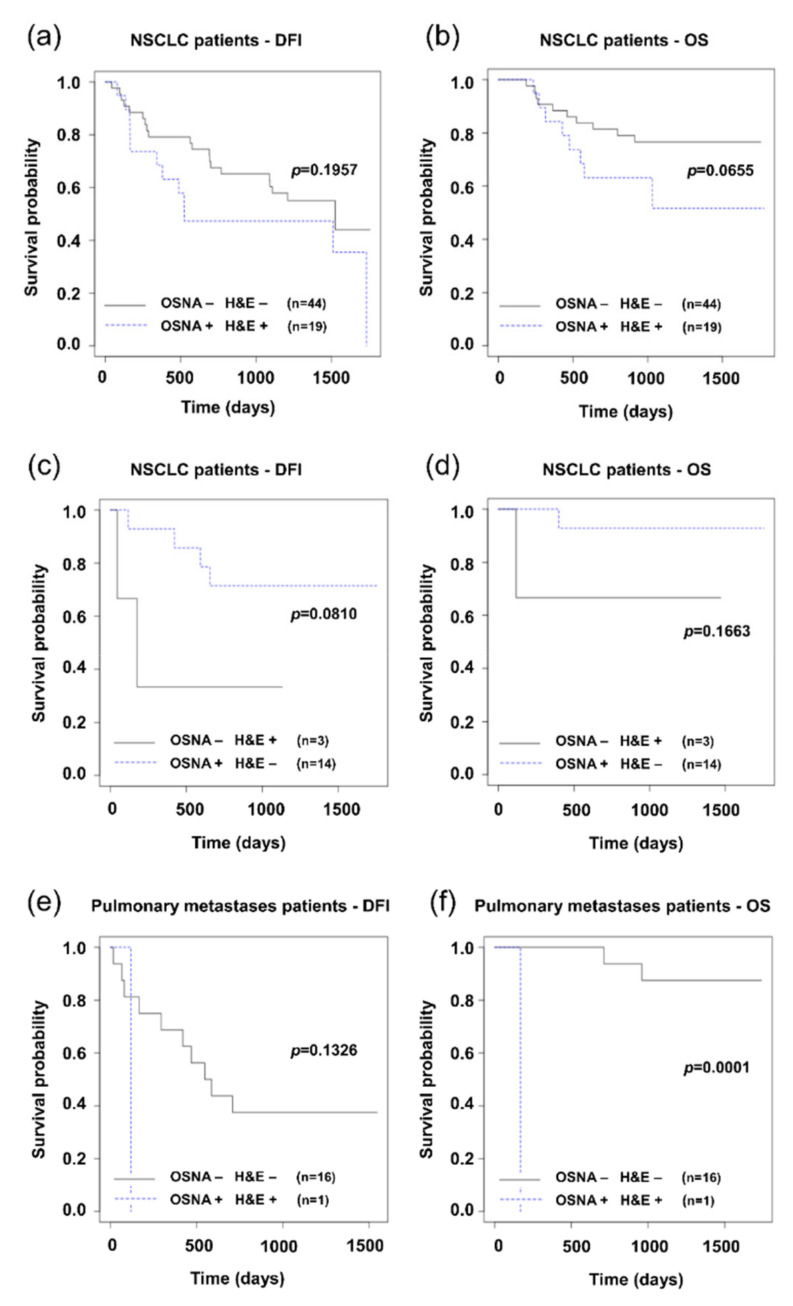
Kaplan–Meier survival distribution functions of patients stratified according to lymph node status (result of method negative: −, result of method positive: +). (**a**) Disease-free survival (DFI) of non-small cell lung carcinoma (NSCLC) patients with concordance of H&E and OSNA (OSNA − H&E − vs. OSNA + H&E +); (**b**) overall survival (OS) of NSCLC patients with concordance of H&E and OSNA; (**c**) disease-free survival (DFI) of NSCLC patients with discordance of H&E and OSNA (OSNA − H&E + vs. OSNA + H&E −); (**d**) overall survival (OS) of NSCLC patients with discordance of H&E and OSNA; (**e**) disease-free survival (DFI) of pulmonary metastases patients with concordance of H&E and OSNA; (**e**) overall survival (OS) of pulmonary metastases patients with concordance of H&E and OSNA.

The clinical significance of the presence of tumor cells in lymph nodes determined by the H&E and OSNA methods was evaluated by analyzing the relation to DFI and OS. The Kaplan–Meier curves comparing NSCLC patients with positive and negative LNs detected concordantly by the H&E and OSNA methods are provided in Figure 2a,b. Survival analysis of patients with pulmonary metastases concordantly analyzed by both methods is shown in Figure 2e,f. The Kaplan–Meier curves show that patients with positive LNs have a shorter OS.

In patients with discordant results (17 out of 80 NSCLC patients), the relevance to prognosis spoke in favor of the H&E method (see Figure 2c). Patients with lymph node positivity as detected by the H&E method and not detected by the OSNA method had a less favorable prognosis (DFI). Here it needs to be noted that the result may be affected by the low number of discordances between the two methods, apparent in particular in the chart for OS, where another limit is the low number of events.

In the clinical use, the status of lymph nodes itself is just a part of the TNM score, based on which the stage of the disease is classified. Table 3 shows the TNM score and stages of the disease in the patients with discordant results of LN examinations by the H&E and OSNA methods.

**Figure 3:**
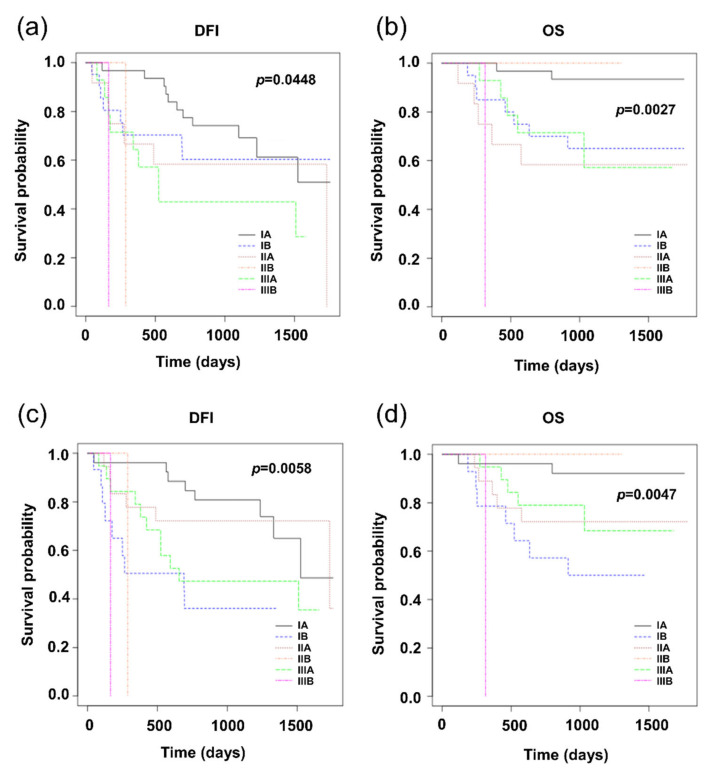
Kaplan–Meier survival distribution functions of NSCLC patients stratified according to stage of disease. (**a**) Disease-free survival (DFI) of patients classified according to the results of H&E; (**b**) overall survival (OS) of patients classified according to the results of H&E; (**c**) disease-free survival (DFI) of patients classified according to the results of OSNA; (**d**) overall survival (OS) of patients classified according to the results of OSNA.

The following charts show the relevance between the stage of the disease based on the TNM score to the prognosis for patients with NSCLC, where the status of the lymph nodes was evaluated either by the H&E method or by the OSNA method (Figure 3).

### 3.4. Proposing a New OSNA Cut-Off Value

**Figure 4:**
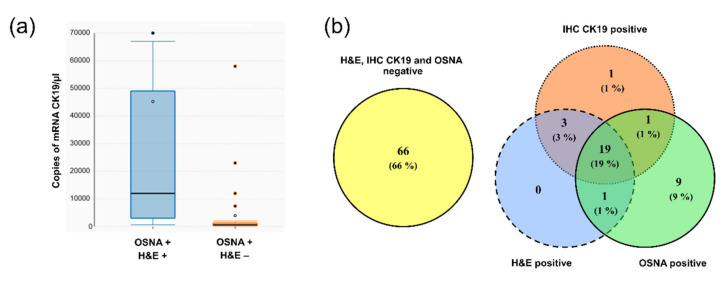
Data supporting new cut-off value. (**a**) Box plots showing copy number of mRNA CK19. Groups of lymph nodes, positive based on both OSNA and H&E methods (OSNA + H&E +), have significantly higher copies of mRNA CK19 than those positive by the OSNA method but negative by H&E (OSNA + H&E −); (**b**) comparison of examination results of patients for the presence of tumor cells using the H&E, IHC CK19 and OSNA methods by Venn diagrams using the new cut-off value of the OSNA method 615 copies of mRNA CK19/µL.

The values of the number of copies of mRNA of CK19 determined in the group of lymph nodes concordantly positive according to the OSNA, and H&E methods were statistically significantly higher (*p* < 0.0001) than in the groups of lymph nodes that were positive only according to the OSNA method (Figure 4a). The relevance of this finding is explained in the discussion.

Based on aforementioned results, we decided to propose a new cut-off value for the OSNA method. This value is based on the number of copies in the specimen with the lowest number of copies that was concordantly positive using the OSNA, H&E, and IHC CK19 methods. In this specimen, the measured expression level of mRNA CK19 was 620 copies of mRNA of CK19/µL. Therefore, we propose that the cut-off value be 615 copies of mRNA CK19/µL. After applying this new cut-off value, 16 groups of lymph nodes detected as positive were revaluated as negative by the OSNA method. The result of revaluation was increase in concordance between the H&E and OSNA methods to 95.1%, when evaluating lymph node groups. When evaluating overall lymph node status of a given patient, the number of patients with a discordant result according to the OSNA and H&E methods decreased from 20 to 13. The complete results are provided in the Venn diagram in Figure 4b. Comparing the prognosis (DFI, OS) of these patients could not be performed correctly due to the low number of discordant patients (13 patients).

## 4. Discussion

The purpose of the study was to compare the three methods for detecting the presence of tumor cells in the lymph nodes of patients with NSCLC and CRC metastases, which is a necessary examination for deciding on further oncological treatment. The clinical relevance of individual methods was assessed based on analyzing the examination result related to the further progress of the disease, i.e., to prognosis (DFI, OS). Determination of tumor cells in the lymph nodes was carried out using the standard method employed by pathologists (preparation staining by H&E), IHC CK19, and by the OSNA molecular biology method. Considering the fact that the results of the IHC CK19 method were concordant with those ascertained by the H&E staining method, with only a single exception, the survival charts include only the comparison of the H&E and OSNA methods. The methodological approach is completely different for H&E and OSNA, in terms of both the quantity of analyzed tissue, and the rate of processing. Therefore, each method has its advantages and disadvantages. It is obvious that evaluation of clinical benefits also depends on priorities. The OSNA method allows the entire lymph node to be analyzed, and compared to the H&E method, it requires less experience from the health care professional.

Compared to certain other tumors, examination of LNs in the case of lung tumors is also specific due to the fact that the concept of the first regional (sentinel) node cannot be applied. One of the reasons is a high percentage of so-called skip metastases [22,23]. This means that it is necessary to remove and examine a higher number of lymph nodes to prove dissemination. In our study, the average number was 14.3 examined LNs per patient. Depending on their anatomical localization, these lymph nodes were pooled into groups according to IASLC mapping scheme. The OSNA method allows lymph nodes belonging to the same group to be pooled and analyzed as a whole.

### 4.1. Concordance of H&E, IHC CK19, and OSNA

Concordance of the H&E and IHC CK19 examinations was ascertained in 99.4% groups of nodes (469 out of 472, see Figure 1), which reflects the similar methodological approach of the two methods. Therefore, in this study, hardly any benefit of IHC CK19 compared to the routine H&E method was observed. This is the reason why we focus below on comparing the OSNA and H&E methods. Out of 472 analyzed groups of LNs, OSNA and H&E provided concordant results in 433 (91.7%) of the groups. OSNA indicated tumor cells in an additional 30 (6.4%) groups of lymph nodes that were H&E-negative.

From the point of view of decisions on treatment, the result specifying the presence of tumor cells in patient LNs is relevant (N-staging). Out of 100 patients, OSNA and H&E were concordant in 80 (80%) of the patients in N-staging; OSNA detected tumor cells in an additional 17 (17%) patients who were H&E-negative. On the other hand, H&E revealed micrometastases in three (3%) patients where the result of the OSNA method was negative. These results are similar to those previously published by other authors. Masai et al. monitored the CK19 expression in primary pulmonary tumors and breast cancer pulmonary metastases. CK19 was detected in 88% of patients with a pulmonary tumor and in nearly 91% of patients with breast cancer metastases [16]. Hayama et al. compared the sensitivity of the OSNA method and H&E examination when examining 40 LNs of 20 patients with primary pulmonary lung cancer (OSNA sensitivity 100%, specificity 92%) [3]. Inoue et al. presented a larger sample when they examined 165 LNs in 49 patients, again with promising results (positive predictive value of the OSNA method 95%, negative predictive value 99%, accuracy 99%) [15]. Nakagawa et al., who examined 410 LNs in 111 patients with NSCLC, found the level of correspondence between the H&E examination and OSNA method to be almost 93%, with sensitivity exceeding 79% [17].

### 4.2. Comparison of H&E and OSNA Based on Relation to Prognosis

From our point of view, the most relevant criterion for correctly detecting tumor cells (the CK 19 expression) for the purposes of N-staging as a part of TNM classification is correspondence with the progress of the disease, vesting the result with clinical significance. The clinical significance of the higher sensitivity of OSNA compared to H&E was assessed based on the analysis of the examination result’s relevance to disease-free interval (DFI) and overall survival (OS). In patients with concordant results detected by both methods (80% of patients), it was observed that positivity of lymph nodes was associated with worse prognosis for both subgroups of patients (NSCLC, pulmonary metastases of CRC). From the point of view of our research aims, patients for whom discordance between H&E and OSNA was observed, were key for further analysis. There were 20 such patients out of 100, 17 with NSCLC and three with pulmonary metastases of CRC. For each patient, 3–5 groups of lymph nodes were examined with differences detected in only 39 out of 472 groups of lymph nodes. It was observed that the further progress of the disease better correlated with the examination based on the H&E method (Figure 2c,d).

To explain this finding, it is necessary to focus on patients with LN positivity detected by H&E but not by OSNA. An explanation could be offered that tumor cells present in LNs of these patients do not express CK19. CK19 is an intermediate filament of the cytoskeleton, which is present in the cells of epithelial origin, but not in LN tissue. The presence of CK19 in an LN is therefore an indicator of the metastatic involvement by tumor cells of epithelial origin [24]. However, some carcinomas in the process of carcinogenesis lose CK19 expression. Such phenomenon was observed in the case of some squamous cell carcinomas [25]. Two out of three H&E-positive but OSNA-negative NSCLC patients really had squamous cell carcinoma as a histological subtype. However, the part of LN examined by pathologist was IHC-CK19-positive. Therefore, a more likely explanation is that tumor cells were present only in the part of lymph node analyzed by pathologist but not in the part processed for OSNA.

Further progress of the disease is affected not only by the positivity of the lymph nodes themselves, but also other parameters assessed by the TNM score, and/or the stage of the disease. More relevant to the progress of the disease is the stage classification based on the N-staging according to results obtained by the H&E method compared to the OSNA method (Figure 3). In this respect, it is necessary to note that patient management (decisions on the administration of adjuvant chemotherapy) was performed in accordance with the results of the routine H&E method, which could definitely have an effect on the comparison results. Kaplan–Meier graphs in Figure 3 raise a question as to whether the adjuvant treatment of stage IB patients is not underestimated.

### 4.3. Proposal of the OSNA New Cut-Off Value

In the OSNA molecular genetic method, the key parameter is the cut-off value of the number of copies of mRNA of CK19. This value determines what number of copies will be analyzed as positive, i.e., interpreted as the presence of tumor cells in the LN. When the study was implemented, the kit for the assay of lung cancer LNs was not yet available and that was why published studies, including ours, employed kits for assays of LNs obtained from patients with breast cancer, including the set cut-off value, which was 250 copies of mRNA of CK19/µL.

The instrument RD100i (and currently available RD210) used for the OSNA method to quantify mRNA of CK19 makes it possible to export the numbers of copies for individual assays. We compared the numbers of copies of mRNA of CK19 for the results concordantly positive with those obtained by the H&E method and for the positive results, which were negative if the H&E method was used. The results concordantly positive with the H&E method had statistically significantly more copies of mRNA of CK19 compared to the OSNA-positive results which were H&E-negative. None of the results concordantly positive with the H&E method had a number of copies lower than 620 copies of mRNA of CK19/µL. In the case of results which were OSNA-positive and H&E-negative, 53% of the specimens had a number of copies lower than 620 copies of mRNA of CK19/µL. The increase in the cut-off value to 615 copies of mRNA of CK19/µL proposed by us would increase the theoretical concordance between the OSNA and H&E methods for the analysis of groups of lymph nodes from 91.7% to 95.1%. With these results reflected in the complete evaluation of the patients’ lymph nodes (N0 vs. N1 or N2), concordance would increase from 80% to 87%.

Studies aiming to define a new cut-off value for the OSNA method better reflecting clinical outcomes have been already published for sentinel lymph node examination in patients with breast cancer [26,27]. Terrenato et al. suggested a cut-off of 2150 CK19 mRNA copies in sentinel lymph node to be a powerful predictor of non-sentinel lymph node positivity to identify patients who really need axillary lymph node dissection [27].

The high value of sensitivity and the cut-off value of the OSNA method are closely related to the clinical relevance of clusters and/or solitary tumor cells, which may or may not have potential for further progression, and/or may be eliminated by the immune system. The study carried out by Ren et al. has proven that the quantity of residual tumor cells in the lymph nodes of patients with bronchogenic adenocarcinoma is relevant to prognosis. The employed method was IHC anti-CK detection, while micrometastases referred to sets of cells—the largest dimension of which was smaller than 2 mm. The authors described that patients with micrometastases had significantly shorter recurrence-free survival and overall survival compared to N0, but significantly longer than for those with N1 macrometastases [28].

We can conclude that for patients who were OSNA-positive and at the same time H&E-negative, the higher sensitivity of OSNA was not reflected in a less favorable progress of the disease. On the other hand, it must be noted that the OSNA method allows the entire lymph node to be analyzed, and, compared to the H&E method, requires less experience from the health care professional. However, the aforementioned advantage is paid by the lower association of OSNA positivity with the progress of the disease (with the cut-off value 250 copies of mRNA of CK19/µL). The increase in the cut-off value to 615 copies of mRNA of CK19/µL increased concordance between the OSNA and H&E methods.

## 5. Conclusions

The results of the study demonstrated a higher percentage of detected micrometastases in hilar and mediastinal lymph nodes when the OSNA method was used for examination. However, the higher sensitivity of the OSNA method in our set of patients corresponded less with the progress of the disease (relevance to DFI, OS) when compared to the H&E method. The less time-consuming OSNA method was associated with an 8.3% discordance of results between OSNA and H&E during the analysis of LNs. The study suggests that an increase in the cut-off value of the OSNA method results in significantly higher concordance between the two methods.

## Figures and Tables

**Table 1:** Characteristics of 80 patients with non-small cell lung carcinoma (NSCLC).

Variables	Number of Patients	%
Gender		
Male	50	62.5
Female	30	37.5
Type of operation		
Pneumonectomy	6	7.5
Bi-lobectomy	7	8.8
Lobectomy	67	83.7
Histology of NSCLC		
Adenocarcinoma	45	56.3
Squamous cell carcinoma	33	41.2
Adenosquamous carcinoma	2	2.5
Pathological T status of NSCLC ^1^		
T1a	20	25.0
T1b	22	27.5
T2a	26	32.5
T2b	6	7.5
T3	5	6.3
T4	1	1.2
Adjuvant chemotherapy		
No	52	65.0
Yes	28	35.0

^1^ TNM scores for malignant tumors, the 7th edition [15] (valid at the time of the patients’ inclusion in the study, 2015–2017).

**Table 2:** Characteristics of 20 patients with pulmonary metastases of colorectal carcinoma.

Variables	Number of Patients	%
Gender		
Male	13	65.0
Female	7	35.0
Adjuvant chemotherapy		
no	12	60.0
yes	8	40.0

**Table 3:** Changes in the pTNM staging depending on H&E, IHC CK19 and OSNA results.

NSCLC (80 Patients)								
Concordance of H&E, IHC CK19 and OSNA assay						61 patients (76.3%)		
Differences between H&E, IHC CK19 and OSNA assay						19 patients (23.7%)		
H&E		IHC CK19		OSNA		Cases	Commentary	
TNM	Stage	TNM	Stage	TNM	Stage			
T2aN0	IB	T2aN1	IIA	T2aN1	IIA	1	higher stage by both OSNA and IHC CK19	
T1aN0	IA	T1aN0	IA	T1aN2	IIIA	2	higher stage by OSNA	
T1bN0	IA	T1bN0	IA	T1bN1	IIA	4		
T1bN0	IA	T1bN0	IA	T1bN2	IIIA	1		
T2aN0	IB	T2aN0	IB	T2aN1	IIA	2		
T2aN0	IB	T2aN0	IB	T2aN2	IIIA	4		
T1aN1	IIA	T1aN1	IIA	T1aN0	IA	1	lower stage by OSNA	
T2aN2	IIIA	T2aN2	IIIA	T2aN0	IB	1		
T1aN2	IIIA	T1aN2	IIIA	T1aN0	IA	1		
T3N2	IIIA	T3N0	IIB	T3N0	IIB	1	lower stage by both OSNA and IHC CK19	
T1bN0	IA	T1bN2	IIIA	T1bN0	IA	1	higher stage by IHC CK19	
**Lung metastases of colorectal carcinoma (20 patients)**								
Concordance of H&E, IHC CK19 and OSNA assay						17 patients (85.0%)		
Differences between H&E, IHC CK19 and OSNA assay						3 patients (15.0%)		
H&E		IHC CK19		OSNA		Cases	Commentary	
N-stage		N-stage		N-stage				
N0		N0		N1		2	higher N-stage by OSNA	
N0		N0		N2		1		

Abbreviations: NSCLC: non-small cell lung carcinoma; H&E: hematoxylin-eosin; IHC CK19: immunohistochemical examination with the anti-cytokeratin 19 antibody; OSNA: One-Step Nucleic Acid Amplification.
